# Theoretical Characterization by Density Functional Theory (DFT) of Delphinidin 3-*O*-Sambubioside and Its Esters Obtained by Chemical Lipophilization

**DOI:** 10.3390/molecules23071587

**Published:** 2018-06-29

**Authors:** Ana Selene Márquez-Rodríguez, Claudia Grajeda-Iglesias, Nora-Aydeé Sánchez-Bojorge, María-Cruz Figueroa-Espinoza, Luz-María Rodríguez-Valdez, María Elena Fuentes-Montero, Erika Salas

**Affiliations:** 1 Facultad de Ciencias Químicas, Universidad Autónoma de Chihuahua, Chihuahua 31125, Mexico; anaselene.marquez@gmail.com (A.S.M.-R.); norasanchez15@gmail.com (N.-A.S.-B.); lmrodrig@uach.mx (L.-M.R.-V.); 2 Technion-Israel Institute of Technology, Haifa 31096, Israel; claugrajeda@gmail.com; 3 Montpellier SupAgro, Montpellier 34060, France; maria-cruz.figueroa@cirad.fr

**Keywords:** anthocyanin, lipophilization, time-depending density functional theory

## Abstract

Anthocyanins are water-soluble phenolic pigments. However, their poor solubility in lipidic media limits their use. This hurdle can be overcome with the lipophilization of anthocyanins, which consists of adding an aliphatic chain to a hydrophilic compound, in order to increase its solubility in lipids. Still, the unspecific chemical lipophilization of anthocyanin-esters produces molecules with different properties from their precursors. In this work, experimental changes of anthocyanin-esters obtained by chemical lipophilization are investigated *in silico* aiming specifically at observing their molecular behavior and comparing it with their anthocyanin precursor. Thus, the analysis of delphinidin 3-*O*-sambubioside and its esters employing Density Functional Theory (DFT) methods, such as the hybrid functional B3LYP in combination with the 6-31++G(d,p) Pople basis set, provides the ground state properties, the local reactivity and the molecular orbitals (MOs) of these compounds. Excited states properties were analyzed by TD-DFT with the B3LYP functional, and the M06 and M06-2X meta-GGA functionals. Local reactivity calculations showed that the electrophilic site for all the anthocyanin-esters was the same as the one for the anthocyanin precursor, however the nucleophilic site changed depending localization of the esterification. TD-DFT results indicate that the place of esterification could change the electronic transitions and the MOs spatial distribution.

## 1. Introduction

**Figure 1:**
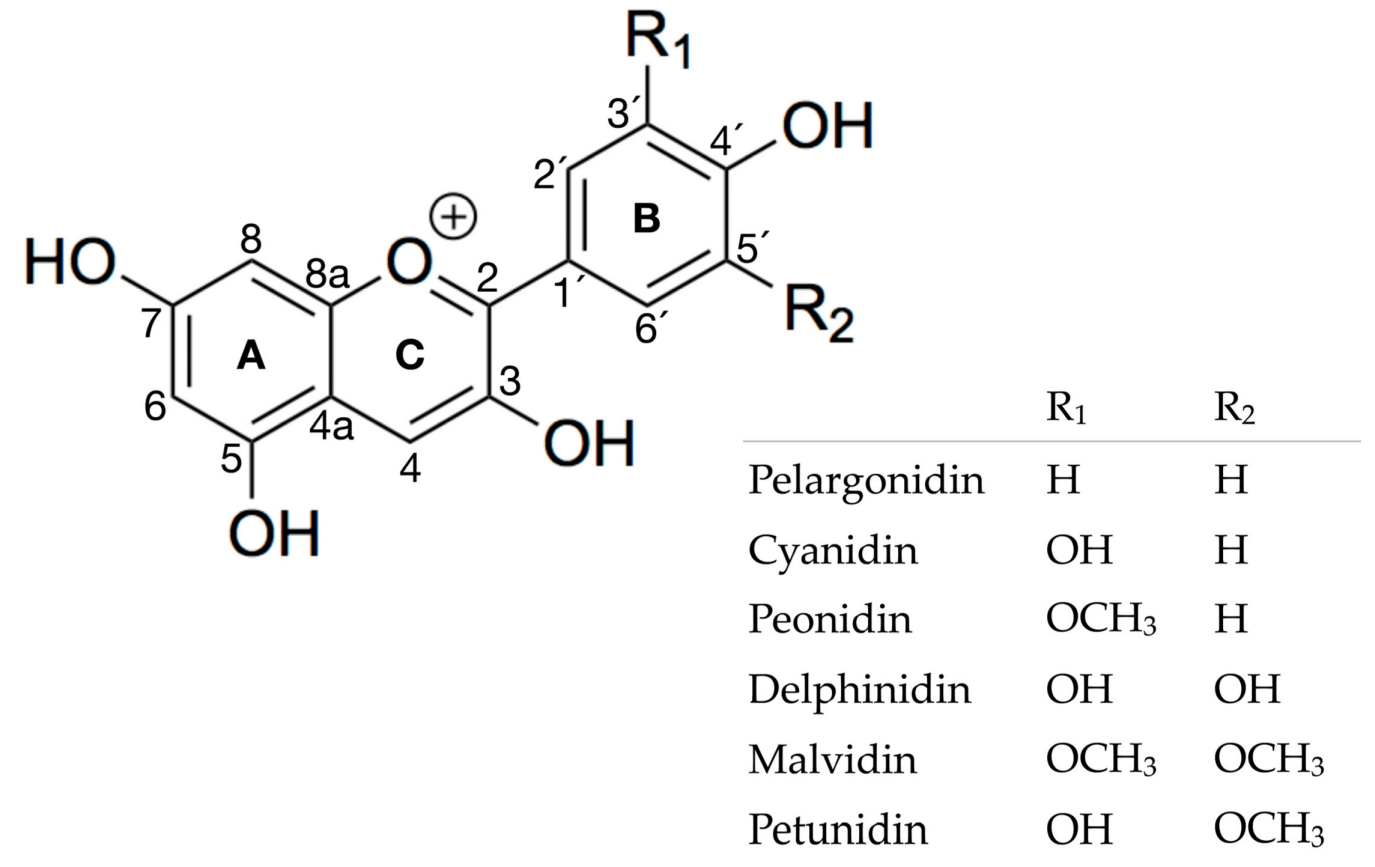
Structure of anthocyanidins: pelargonidin, cyanidin, peonidin, delphinidin, malvidin and petunidin.

Anthocyanins are phenolic compounds from the flavonoids family, which are produced naturally in flowers and fruits (Figure 1). These water-soluble pigments change color in aqueous solution from red to blue depending on the pH. This spectrum responds to the change in the form of the molecule’s structure when it gets protonated or hydrated, thus in aqueous solution different forms co-exist in equilibrium [1]. Besides the equilibrium states, anthocyanin studies have been performed to determine their reactivity, molecular interactions, antioxidant properties, among others [2,3]. 

The application of anthocyanins as industrial pigments has been limited due to their poor solubility in lipidic media. To overcome this solubility issue the lipophilization of anthocyanins has successfully been performed by enzymatic and chemical approaches [4,5]. The lipophilization reaction consists of adding an aliphatic chain to the hydrophilic anthocyanin, in order to change the polarity in the molecule and favor its solubility in non-polar matrixes. For example, a recently reported enzymatic lipophilization of malvidin 3-*O*-glucoside with oleic acid and *Candida antarctica* lipase B as catalyst generated a single anthocyanin-ester with attractive color characteristics [4]. In a different study [5], a chemical reaction was performed with delphinidin 3-*O*-sambubioside (DpS) and octanoyl chloride in an acidic medium; the reaction produced mono-, di- and tri-esters which could not be purified and fully characterized by experimental methods. 

Some studies have been supported by theoretical research, which have incorporated geometrical analysis, molecular interactions, antioxidant properties, optical properties and solvation effects, to name a few [6,7,8]. These models allow the explaining and understanding of the behavior of the experimental phenomena at a molecular level, converting in silico studies into an important tool, which provides significant information about anthocyanins [9]. Particularly, the optical features of anthocyanins have been calculated with semi-empirical [10], Hartree-Fock (HF) [11], and Density Functional Theory (DFT) [12] methodologies employing several functionals and basis sets. The excited states obtained by HF have used the single excitation–configuration interaction (SE–CI) calculation to describe the electronic characteristics of anthocyanins after the geometry optimization by DFT methodology, employing the B3LYP hybrid functional and the D95 basis set [6]. Concerning semi-empirical methods, the Zerner’s Intermediate Neglect of Differential Overlap (ZINDO) [13] and the Austin Model 1 (AM1) [14] showed to correctly describe the maximum wavelength (λ_max_) of anthocyanins [15]. However, DFT is one of the electronic structure most used methods to calculate anthocyanins theoretical features, whereas Time-Depending DFT [16] has provided more accuracy to represent UV-Vis absorption spectra [6,17,18].

Specifically, the TD-DFT methodology approaches the experimental λ_max_ for flavylium cations, however, optical properties calculated employing pure DFT functionals (BLYP, BP86, PBE) underestimate the main absorption band, in accordance with Anouar et al. [18]. The hybrid functionals, which include HF exchange contributions and have given better results, are the B3LYP, B3P86, PBE0 or the meta-GGA functionals [18,19,20]. Moreover, diffuse functions, polarized functions for heavy atoms, and the diffuse functions for hydrogen atoms (for example the 6-31+G(d,p) Pople basis set) have been suggested when analyzing electronic properties of anthocyanins [9]. 

The solvation effects have been analyzed by the application of the polarizable continuum model (PCM) using the integral equation formalism polarizable variant (IEFPCM) and water as solvent, which describes the contribution of the solvent in theoretical studies of anthocyanins [21]. However, the full characterization of anthocyanin color has not yet been achieved by a theoretical methodology. Based on a previous work [19] the B3LYP [22], M06 and M06-2X [23] were chosen for this study; these functionals have a 20%, 27% and 54% of HF exchange contribution, respectively. Therefore, the aim of this work is to explore the electronic and optical properties of the delphinidin 3-*O*-sambubioside and its chemically obtained esters, employing different DFT approaches.

## 2. Results and Discussion

### 2.1. Experimental Results

**Scheme 1:**
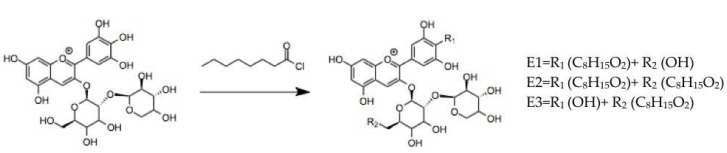
Lipophilization reaction of DpS. Ester 1 (E1) is a monoester with the octanoyl chain joined to the B ring; Ester 2 (E2) is a di-ester with the octanoyl chains joined to the B ring and the sambubioside; Ester 3 (E3) is the monoester with the octanoyl chain joined to the sambubioside.

**Figure 2:**
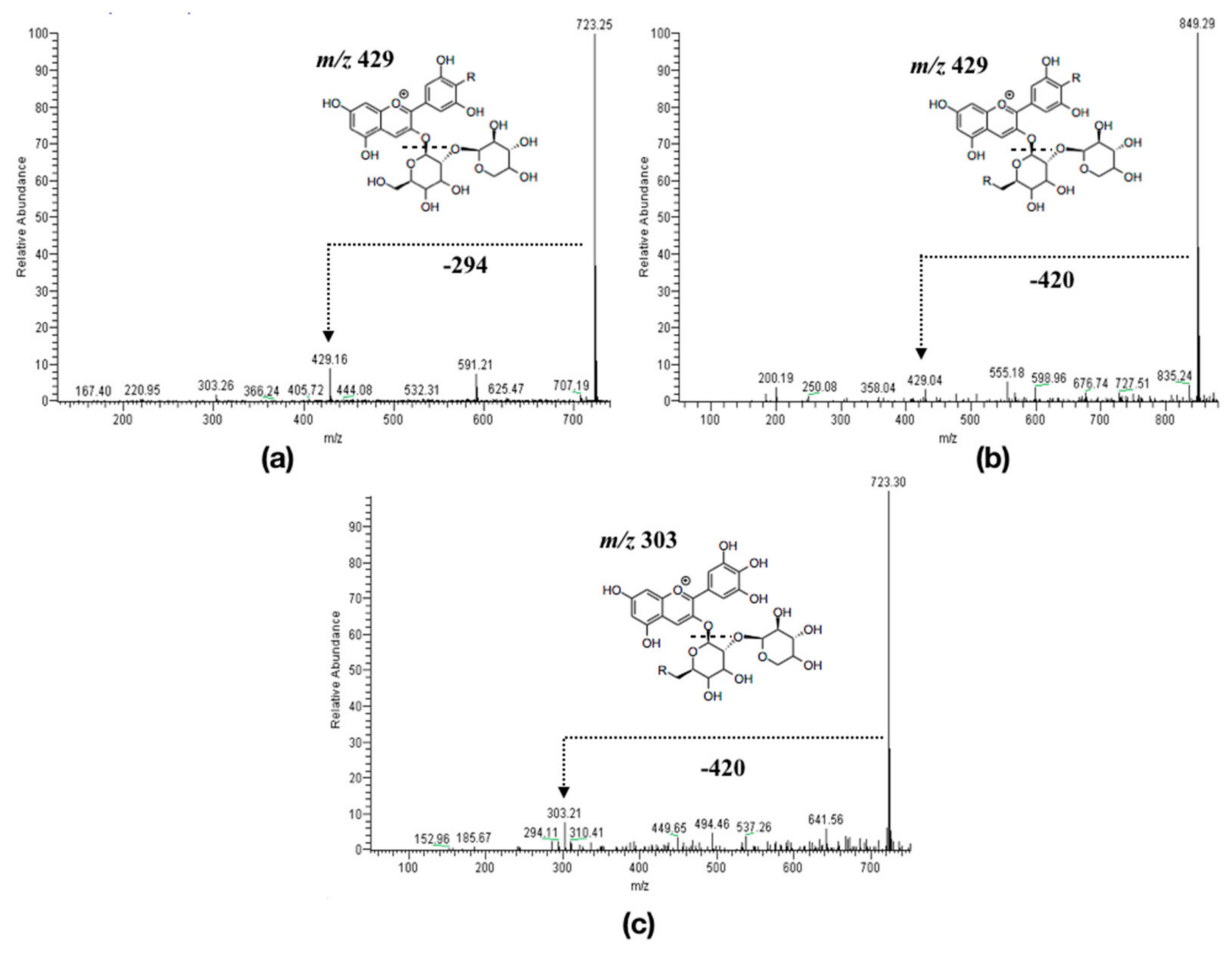
Full-scan (+) ESI of: (**a**) the monoester E; (**b**) di-ester E2 and (**c**) monoester E3.

The lipophilization of anthocyanins has only been achieved recently in three studies, via chemical or enzymatic reactions [4,5,24]. The lipophilization of DpS was successfully carried out employing octanoyl chloride as reported by Grajeda-Iglesias [5]. The anthocyanins were extracted from *Hibiscus sabdariffa* calyxes and then purified; the anthocyanins were used for the lipophilization reaction that is described in the methodology section of this article. The products of this reaction were two different monoesters (E1 and E3) and a di-ester (E2). The positions where the esterification can take place are shown in Scheme 1. These structures were partially elucidated using HPLC/MS/MS. Fragmentation pathways in MS/MS spectrometry disclosed that the esterification of the anthocyanin occurred either in the sambubioside unit, in the flavylium unit or both (Figure 2). It shows the full-scan ESI in positive mode of the esters obtained by chemical lipophilization. In Figure 2a two peaks can be observed, the peak at *m*/*z* 723 corresponds to the base peak of the delphinidin 3-*O*-sambubioside monoester (E1), and the peak at *m*/*z* 429 corresponds to the flavylium fragment plus the aliphatic chain, while the loss of *m*/*z* 294 indicates the sambubioside. Thus, it could be concluded that the lipophilization took place in the flavylium core. Then, the second monoester (E3) is shown in Figure 2c, where the base peak is displayed at *m*/*z* 723, while the *m*/*z* 303 indicates the presence of the anthocyanin´s aglycon. Thereby the loss of *m*/*z* 420 corresponds to the mass of sambubioside and the octanoyl chain, which evidences that the lipophilization was carried out in the sambubioside fragment. Finally, the delphinidin 3-*O*-sambubioside di-ester (E2) can be observed at *m*/*z* 849 corresponding to its base peak, while the peak at *m*/*z* 429 implies that the mass of the flavylium core is esterified; thus, the loss of *m*/*z* 429 also denotes that the sambubioside was esterified (Figure 2b). These esters suffered a hypochromic effect (shown in the inserts of Figure 4a–c), where the more significant loss of intensity at 520 nm was in the di-ester.

It has been indicated that a loss of protons in the anthocyanin structure could produce a hypochromic effect [25,26]. Besides, the change of color in anthocyanidins depends on the grade of substitution of -OH, -OCH3 and glycosylation. These phenomena have been experimentally observed and theoretically analyzed [18,27]. Thus, the union of a chain to the flavylium core could induce the hypochromic effect. Cruz et al. [4] also saw a hypochromic effect when the enzymatic lipophilization of malvidin 3-*O*-glucoside occurred in the glucose unit. The ester obtained from lipophilization of malvidin 3-*O*-glucoside with oleic acid and *Candida antarctica* lipase B had a hypochromic and bathochromic effect as well, but the new pigment preserved its attractive color, contrasting with the chemical lipophilization of DpS. This effect on the color intensity in the enzymatic produced anthocyanin ester could be due to the position of the esterification, which occurred at the glucose moiety of the malvidin. Meanwhile, the chemical lipophilization is not selective and the hypochromic effect is noticeable when the esterification occurs in the flavylium core.

### 2.2. Theoretical Results

#### 2.2.1. Theoretical Methodology Selection

Only a small number of atomic structures of phenolic compounds, with exact bond length and angles, have been reported. X-Ray diffraction (XRD) data for cyanidin bromide [28] is one of them. Therefore, this experimental XRD structure was used to test the quality of the approximations for each functional. In this work, the optimized geometrical structure of Cyanidin was calculated with the DFT B3LYP [22], M06-2X and M06 [23] functionals with the 6-31G++(d,p) [29] basis set. The results from the regression analysis are shown in Table 1. The highest correlation coefficient (R^2^) corresponds to the B3LYP/6-31++G(d,p) level of theory, which coincides with those reported by Sanchez-Bojorge et al. in 2015 [19].

Based on this analysis for Cyanidin, the methodology B3LYP/6-31G++(d,p) was used for the geometry optimization in DpS, E1, E2 and E3 molecules. The frequency analysis was performed to find the global minimal of the potential energy hypersurface and confirm the absence of imaginary frequencies. Thus, in order to start the Self-Consistent Field (SCF) calculations from the structural configuration closest to the global minimum energy, the ground state was obtained first in gas phase. Afterwards, the geometry optimizations and frequency analysis were done in aqueous phase, employing the IEFPCM method with the same level of theory for all the compounds. No imaginary frequencies were found in the final theoretical structures of the DpS and its esters.

#### 2.2.2. Reactivity Analyses

**Figure 3:**
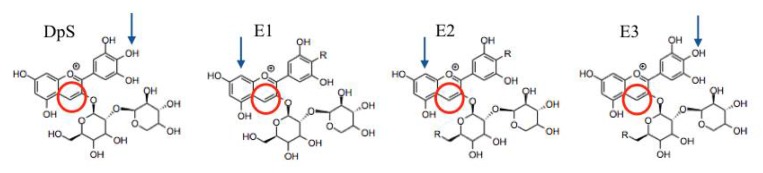
Local reactivity sites for anthocyanins and its esters calculated with the B3LYP/6-31++G(d,p) methodology. The red circle (C4) represents an electrophilic site and the blue arrow (O4′ or C8) represents a nucleophilic site.

Chemical reactivity calculations were done in aqueous phase for all the molecules with the B3LYP/6-31++G(d,p) level of theory. To model the anthocyanin in pH < 2, the Fukui functions were calculated for the flavylium form of the DpS. The results for the DpS and its esters show the site where an electrophilic or nucleophilic attack could occur. Only one of the reactions can happen at a time. The nucleophilic attack will happen at the electrophilic site of the molecule and vice versa for the electrophilic attack. Thus, a nucleophilic molecule, like water, will attack the electrophilic site of the anthocyanin. The reaction of esterification, a form of electrophilic attack, will take place in the nucleophilic site of the anthocyanin. DpS local reactivity is located at C4 and O4′, the electrophilic and nucleophilic sites, respectively (Figure 3).

The structure of E1 is based on the nucleophilic reactivity site of DpS. The esterification of DpS was done in the O4′. The reactivity calculations for E1 give the same electrophilic site (C4), but the nucleophilic site changed to C8. However, a second esterification of E1 at C8 is not possible due to the lack of oxygen, but it can take place at O7, which is the second nucleophilic site.

The geometry for E2 was chosen in accordance with experimental data obtained from LC/MS/MS (see Figure 3) and the reactivity calculations took into account the following facts. The mass spectrometry shows a di-ester: one ester where the octanoyl chain was joined to the flavylium moiety, and the other one in the sambubioside unit. The reactivity for E2 is presented in the C4 for the electrophilic site, and in C8 for the nucleophilic site. Similarly to E1, a second esterification cannot take place in the C8, instead it could take place at O7 or O5. The modeling for a di-ester with one chain joined to the B ring and another chain joined to the A ring was not calculated, in agreement with the experimental data.

The geometrical model for E3 was also chosen in accordance with mass spectrometry data, where it was observed that the octanoyl chain was joined to the sambubioside part. For this ester, the electrophilic and nucleophilic sites were in the C4 and O4′, respectively. The reactivity for the E3 was equal to the native anthocyanin DpS.

The electrophilic site for the DpS, in the C4 of the C ring, agrees with the experimental results; whereas the principal nucleophilic site (O4′) for the DpS does not match with previously reported data by Fulcrand [2] and de Freitas and Mateus [30] at the C6 or C8 of the A ring. In summary, the electrophilic site of all anthocyanin-esters remained in the position C4 of the C ring. Though, for E1 and E2, the nucleophilic site changed to the C8 in the A ring and for E3 it remained in the O4′ of the B ring. 

#### 2.2.3. Excited States

Regarding the molecular structure modification, the change in the absorption spectra was analyzed in a theoretical study of pyranoanthocyanins [31] showing that the site of deprotonation could change the theoretical UV-Vis spectra. Thus, in DpS, the position where the hydrogen atom was substituted by the aliphatic chain also produces different spectra for the anthocyanin-esters. Thereby, the comparison of various theoretical spectra obtained with different functionals in this study will give qualitative insights into how the position of the lipophilization affects the absorbance of the molecule.

The excited states for DpS were calculated with the 6-31++G(d,p) basis set and the B3LYP, M06 and M06-2X functionals, employing the IEFPCM formalism in aqueous phase, where the calculated maximum wavelength (λ_calc_) for each methodology were 487.7, 479.9 and 436.9 nm respectively. All the employed functionals presented a hypsochromic shift in their λ_calc_ for all the analyzed esters, when compared with the maximum absorption wavelength (λ_calc_) of the DpS. The differences at λ_exp_ = 526 nm absorbance between the DpS and its esters encouraged the detailed comparative study of the predictive capabilities of each functional, within the TD-DFT methodology, which is presented below. A detailed description of previous functionals will be discussed in this section. The λ_calc_ closest to the experimental λ_exp_ value registered at 526 nm for the DpS were obtained with the B3LYP functional, which has a 20% HF exchange. In this manner, Table 2 shows the most important theoretical results for electronic transitions and their percentage of contribution, the corresponding vertical absorption wavelengths (λ_calc_) and oscillator strengths (*f*) of the four analyzed molecules calculated particularly with the DFT: B3LYP/6-31++G(d,p) methodology.

**Figure 4:**
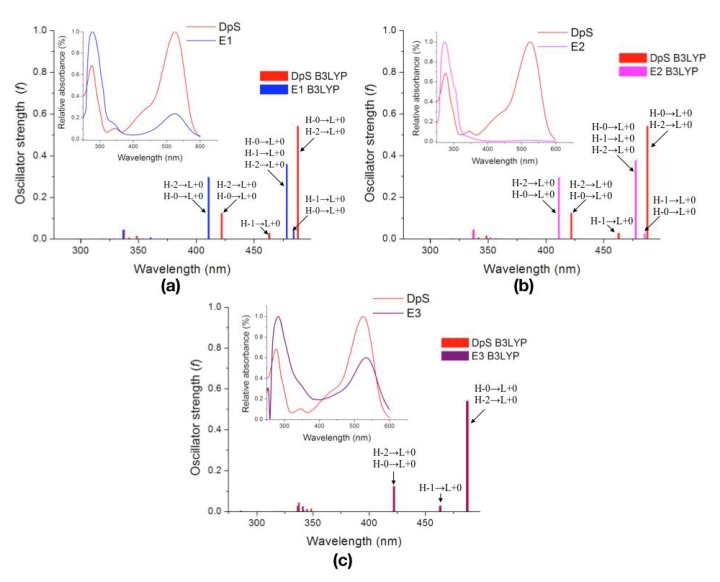
UV-Vis absorption spectra calculated with TD-DFT: B3LYP/6-31++G(d,p) of the DpS compared with the: (**a**) monoester E1; (**b**) di-ester E2; (**c**) monoester E3. [The inserts present the experimental UV-Vis spectra of the DpS contrasted with E1 (**a**), E2 (**b**) and E3 (**c**)].

Figure 4 shows the experimental and theoretical absorption spectra of DpS compared with E1, E2 and E3 at B3LYP/6-31++G(d,p) level of theory. It can be seen, that the normalized TD-DFT response of the oscillators of the native anthocyanin and its esters show UV-Vis differences; which are similar to the experimental response, for example, the absorbance intensity is diminished when an esterification has occurred. Although, the difference in the relative absorbances cannot be quantified, as the experimental UV-Vis spectra of DpS and its different esters were analyzed at different concentrations.

The main experimental absorption band for DpS, which is around 520 nm, was assigned to the first excited state that corresponds to the theoretical H-0→L+0 and H-2→L+0 electronic transitions at 487.7 nm (*f =* 0.5409) (see Figure 4). The experimental UV-Vis spectrum of DpS presented a shoulder at 420 nm; it was assigned to the third excited state at 422 nm (*f =* 0.5391) and related to H-2→L+0 and H-0→L+0 electronic transitions.

When a lipophilization occurs in the sambubioside fragment (E3), the behavior in the theoretical UV-Vis spectrum is similar to the DpS spectrum, however, experimentally a minor hypochromic effect is observed in the anthocyanin-ester (See insert in Figure 4c). As mentioned before, a decrease in intensity of the absorbance was observed in the Malvidin 3-*O*-glucoside oleic acid conjugate reported by Cruz et al. in 2016 [4], with no loss of the color attractiveness.

E1 and E2 molecules display a different behavior compared with DpS and E3, although theoretically similar between them. It is noticed that for E1 the experimental band at 520 nm corresponds to two excited states, where the first state is mainly described by the H-1→L+0 electronic transition at 484 nm (*f =* 0.0417). Meanwhile, for E2, the same experimental band disappears from the spectrum (see insert in Figure 4b), but the theoretical calculations showed an excited state at 485.4 nm (*f =* 0.0265) that mainly corresponds to the H-1→L+0 electronic transition. In the second excited state for E1 and E2, electronic transitions are mainly given by H-0→L+0 at 478.1 nm (*f =* 0.3598) and 477.6 nm (*f =* 0.3769) respectively. While, for E1 and E2, the excited states corresponding to the absorbance at 420 nm are related to the H-2→L+0 and H-0→L+0 electronic transitions at 410 (*f =* 0.2969) and 411.2 nm (*f =* 0.2943), respectively.

For the DpS and E3, the TD-DFT results show that the H-0→L+0 transition is the main contribution of the first excited state, in accordance with Woodford [32]. Nevertheless, using the B3LYP functional for the E1 and E2 a different configuration arises: the first excited state is represented mainly by the H-1→L+0 electronic transition, and the second excited state, is given by H-0→L+0 (the main electronic transition), which corresponds to the strongest oscillator force. This inversion could be explained when lipophilization occurs in the flavylium core, causing a hypochromic shift. Moreover, E1 and E2 second excited states present an increment from two to three electronic transitions, while E3 maintained only two transitions, as happened with DpS.

Other two functionals were tested in order to corroborate the trend of the obtained electronic transitions calculated by B3LYP. The M06 functional was one of them. It presented the first excited state for the DpS at 479.9 nm, which is 46 nm below the λ_exp_ = 526 nm (Table 3). The first excited state is associated with the H-0→L+0 and H-2→L+0 electronic transitions, with an oscillator strength value of 0.5886. Second and third excited states show equal electronic transitions than the ones obtained when the B3LYP functional was employed.

**Figure 5:**
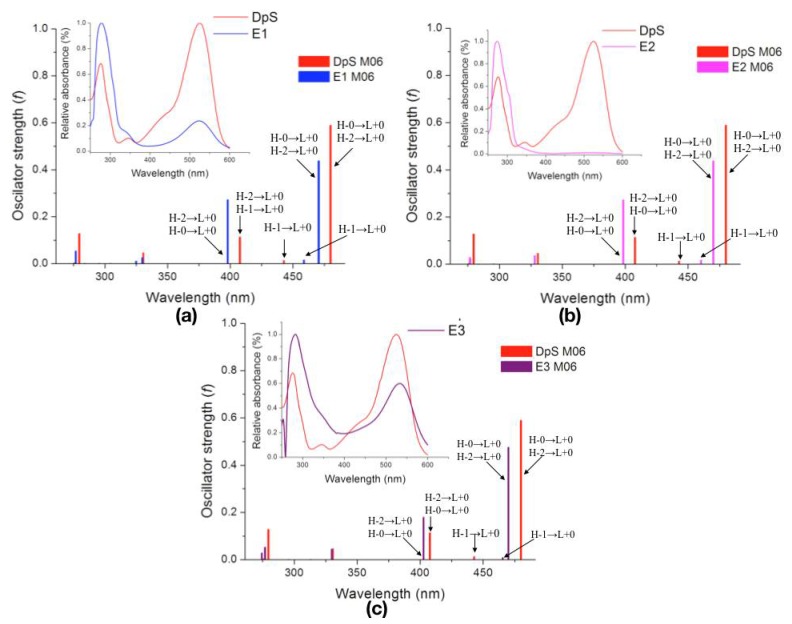
UV-Vis absorption spectra calculated with TD-DFT: M06/6-31++G(d,p) of the DpS compared with the: (**a**) monoester E1; (**b**) di-ester E2; (**c**) the monoester E3. [The inserts present the experimental UV-Vis spectra of the DpS contrasted with E1 (**a**), E2 (**b**) and E3 (**c**)].

The M06 functional, with a 27% of HF exchange, does not show the inversion in the excited states presented by B3LYP functional between the first and second excited states for E1 and E2. Thus, the M06 functional presents only a hypsochromic shift in the λ_calc_ when the esterification occurs either in the flavylium moiety or in the sambubioside unit, without any other outstanding changes in the electronic transitions (Figure 5). The other functional used for comparison with B3LYP was M06-2X. Table 4 shows the relevant theoretical information about the electronic transitions, the calculated vertical absorption wavelengths and the oscillator strength at M06-2X/6-31++G(d,p).

**Figure 6:**
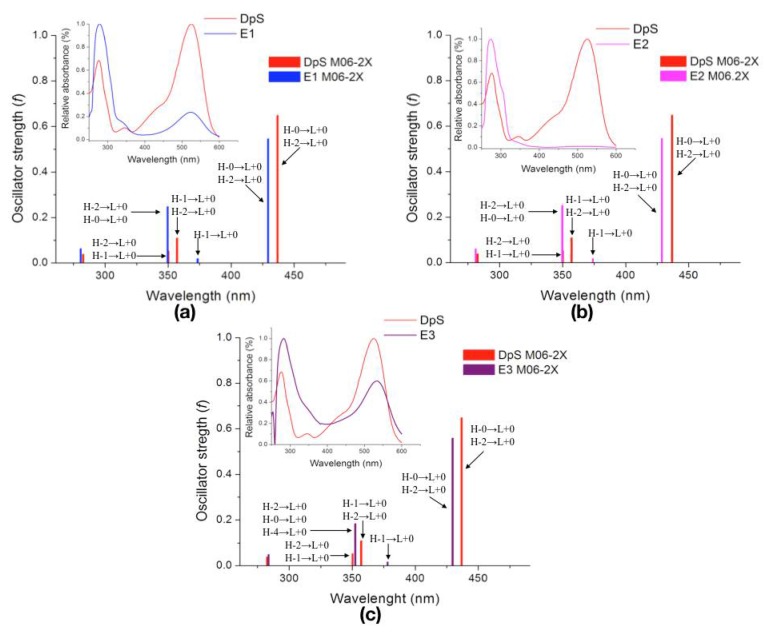
UV-Vis absorption spectra calculated with TD-DFT: M06-2X/6-31++G(d,p) of the DpS compared with the: (**a**) monoester E1; (**b**) di-ester E2; (**c**) the monoester E3. [The inserts present the experimental UV-Vis spectra of the DpS contrasted with E1 (**a**), E2 (**b**) and E3 (**c**)].

The first excited state for DpS calculated with M06-2X/6-31++G(d,p) level of theory is assigned to H-0→L+0 and H-2→L+0 electronic transitions at 436.9 nm (*f =* 0.6474), which means a severe shift from λ_exp_ = 526 nm (Figure 6). This difference in maximum absorption wavelength can be attributed to the HF exchange of the M06-2X functional (54%), which is twice the amount compared to the B3LYP or the M06 functionals. The second excited state, corresponding to the experimental band at 420 nm, is assigned to H-1→L+0 and H-2→L+0 electronic transitions, displayed at 357.2 nm (*f =* 0.1095), and it also still shows a shift from the experimental value.

Based on the results reported in Table 4, when the anthocyanin-ester presents an esterification, either in the flavylium core (E1), in the sambubioside (E3) or both (flavylium core and sambubioside) (E2), the first excited state preserves the same electronic transition as the DpS; while the oscillator strength displays a diminished value compared with the DpS at same level of theory. The second excited state for E1, E2 and E3 presents only the H-1→L+0 electronic transition at 373.5 (*f* = 0.0188), 374 (*f* = 0.0182) and 378.1 nm (*f* = 0.0161) respectively.

The diminishing in the oscillator strength of the anthocyanin-esters is noticeable for all functionals, compared with the oscillator strength of the DpS. This marked effect is not observed for E3 when it is calculated with B3LYP/6-31++G(d,p) level of theory, where the oscillator strength and the λ_calc_ remained similar to the values of DpS.

In summary, the results of M06-2X are similar to the ones of M06 functional, and the results of both meta-GGA are different from B3LYP, which presented an inversion of excited states. In addition, the B3LYP functional has been reported [19] to correlate better with the experimental data for the absorption properties. 

The B3LYP functional explained better the UV-Vis spectra than Minnesota functionals, since the HF contribution is only 20% for B3LYP [33]. This implies that the LYP correlation energy considered in the B3LYP functional, which is partially responsible for the covalent and conjugated nature of our molecules, adjusts better than the one from the M06 family (where HF exchange are between 27% and 54%) [23]. This result coincides with the one reported by Anouar [18], where the B3P86 and B3LYP functionals show the best performance for phenolic compounds.

#### 2.2.4. Molecular Orbitals

In order to gain insight into the origin of the differences between the esterified molecules, the B3LYP/6-31++G(d,p) methodology (the one which gave a structure nearest to experimental XRD data) was employed to perform the following molecular orbitals (MOs) calculations. 

**Figure 7:**
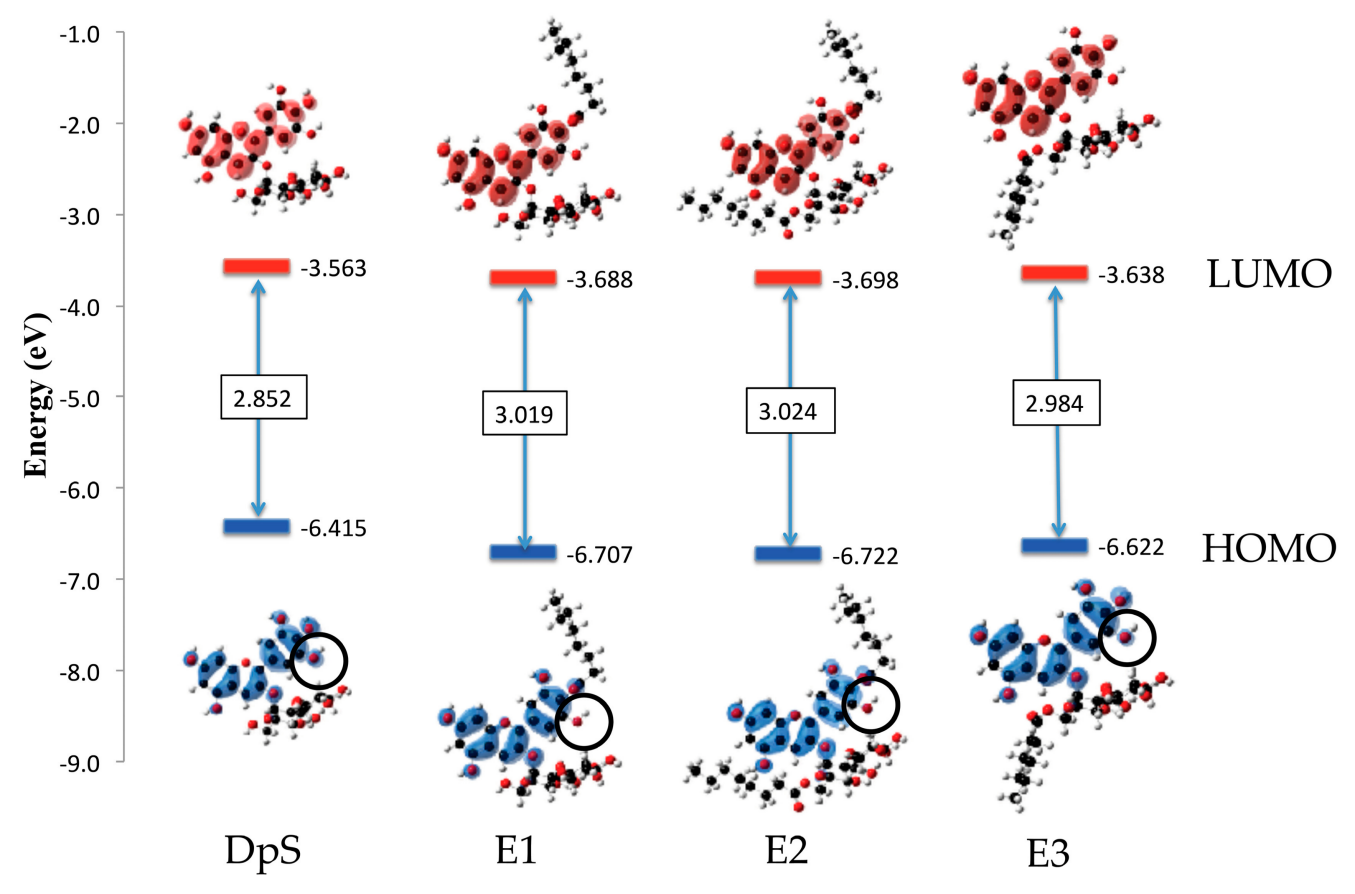
Molecular orbitals and HOMO-LUMO gaps for delphinidin 3-*O*-sambubioside and its esters. The black circles highlight the position where the HOMO is affected by the esterification at ring B.

Highest occupied molecular orbital (HOMO) and lowest unoccupied molecular orbital (LUMO) maps corresponding to the principal electronic transition (H-0→L+0) were estimated through a single point calculation. Computed MOs are all distributed along the anthocyanin structures, as shown in Figure 7. It seems there are no significant differences between MOs of the DpS molecule and its esters. However, there is a slight dissimilarity in their spatial distributions, whereas DpS and E3 exhibit some electronic density at O5′, E1 and E2 do not (Figure 7). The HOMO-LUMO gap for the DpS (2.852 eV) is similar to the one mentioned by Rustioni [34] (2.87 eV). 

The HOMO-LUMO gaps are also almost equal in all the analyzed structures. Although, when the esterification occurs in the sambubioside unit (E3), its HOMO-LUMO energy gap is close to the one of DpS.

Comparing these findings with the previous reactivity results, it can be added that the electrophilic sites for all the studied molecules in this work correspond to the site of the largest LUMO density, and the nucleophilic sites are related to the largest HOMO density. Thus, although no significant differences are shown in the calculated MOs for DpS and E3, these present different nucleophilic behaviors than E1 or E2, which were esterified in the B ring. Chemical reactivity plus MOs analysis might clarify the fact that, any insignificant change occurring in the principal electronic transitions could modify the macroscopic properties of the molecules.

## 3. Materials and Methods

### 3.1. Experimental Methodology

#### 3.1.1. Anthocyanin Extraction and Purification

The purification of the hibiscus anthocyanins was described by Grajeda-Iglesias [35]. Briefly, 100 g of grounded powder of *Hibiscus sabdariffa* L. was mixed with 250 mL of acidified water-ethanol (80–20, *v*/*v*) and then placed in a cold ultrasonic bath for 30 min. The obtained extract was then filtered, freeze-dried and stored at −20 °C. The fractionation of the phenolic extract was carried out by solid phase extraction (SPE) using a Sep-Pak^®^ cartridges (35 cc vac, 10 g, 55–105 μm; Millipore Waters, Milford, MA, USA); 200 mg of the hibiscus extract was dissolved in 2 mL of acidified water (acetic acid 5%, *v*/*v*) and then placed on a cartridge previously activated with methanol and equilibrated with acidified water. The elution was done by increasing the percentage of methanol in an alcohol-water mixture. Two main fractions were obtained from the SPE, the fraction 2 (F2) containing DpS and fraction 3 (F3) containing cyanidin 3-*O*-sambubioside. Each fraction obtained was evaporated under vacuum at 32 °C and freeze-dried for further purification. Semipreparative HPLC was used to purify delphinidin 3-*O*-sambubioside. ^1^H-NMR (400 MHz) and ^13^C-NMR analysis were performed to confirm structure and purity [35].

#### 3.1.2. Anthocyanins Lipophilization

Lipophilization of the purified DpS was performed following the experimental procedure described in [5]. Briefly, a purified fraction of DpS (0.0168 mmol equiv) was completely dissolved in 1 mL of DMF. Ten equivalents of octanoyl chloride were incorporated into the anthocyanin solution; the reaction was left for 24 h at room temperature in an inert atmosphere in the dark. The reaction progress was monitored on HPLC (HPLC-ESI-MS, Thermo Fisher, San Jose, CA, USA), and it was stopped adding the solution into a Sep-Pak C18, previously activated with methanol and equilibrated with acidified water. Three different fractions were obtained by sequentially adding acidified water, ethyl acetate and methanol. The fractions were rotaevaporated until dryness and recovered in methanol-acidified water (50–50 *v*/*v*).

### 3.2. Theoretical Methodology

#### 3.2.1. Methodology Selection

The hybrid DFT [12] functionals B3LYP [22,33,36], M06 and M06-2X [23] with the 6-31G++(d,p) [29] basis set were employed to find the best correlation between theoretical data of cyanidin and X-ray diffraction (XRD) data of cyanidin bromide [28]. The best correlation between experimental and theoretical methodologies was used to select the method for obtaining the geometrical structure of the DpS. The selected method B3LYP/6-31++G(d,p) was employed for the geometrical structure optimization of delphinidin 3-*O*-Sambubioside (DpS), delphinidin 3-*O*-sambubioside monoester (E1), delphinidin 3-*O*-sambubioside di-ester (E2) and delphinidin 3-*O*-sambubioside monoester (E3), shown in Figure 1. The optimized parameters of ground state structure of each compound were used to calculate analytical frequencies at the same level of theory, where was confirmed the absence of imaginary frequencies.

#### 3.2.2. Local Reactivity

Fukui functions $f_{k}\left(r\right)$ given in Equation (1) [37] were employed to estimate the local reactivity in the DpS, E1, E2 and E3 molecules. Fukui functions for specific atom *k* are computed by:

(1) $$f_{k}\left(r\right)=f_{k}^{+}\left(r\right)+f_{k}^{-}\left(r\right),$$

(2) $$f_{k}^{+}\left(r\right)=q_{k}\left(N+1\right)-q_{k}\left(N\right),$$

(3) $$f_{k}^{-}\left(r\right)=q_{k}\left(N\right)-q_{k}\left(N-1\right),$$

where: $f_{k}^{+}\left(r\right)$ is the electrophilic contribution (Equation (2)) and $f_{k}^{-}\left(r\right)$ is the nucleophilic contribution (Equation (3)).

B3LYP/6-31++G(d,p) level of theory was employed with the IEFPCM [38] in aqueous phase to obtain the Hirshfeld population analysis and finding the electrophilic and nucleophilic sites of all molecules. 

#### 3.2.3. Excited States

Geometry structure of anthocyanins and its esters were obtained employing the B3LYP/l6-31++G(d,p) methodology, calculated first in gas phase, through the approximation of an isolated molecule; while the solvent effect was determined with the polarizable continuum model (PCM), using the IEFPCM formalism in aqueous phase. Then, the excited states of anthocyanins and its esters were obtained by TD-DFT [16], using the B3LYP, M06 and M06-2X functionals with the 6-31++G(d,p) basis set, in order to calculate the UV-Vis spectra. The time-dependent equations were solved for ten excited states and employing the IEFPCM model in water to find the solvent effect for each molecule.

#### 3.2.4. Molecular Orbitals

The HOMO (High occupied molecular orbital) energy and LUMO (Lower unoccupied molecular orbital) energy and the HOMO-LUMO energy gap for each pigment were obtained from a single point calculation for the ground state, employing the IEFPCM model in aqueous phase. B3LYP/6-31++G(d,p) level of theory was used for MOs calculations.

**Figure 8:**
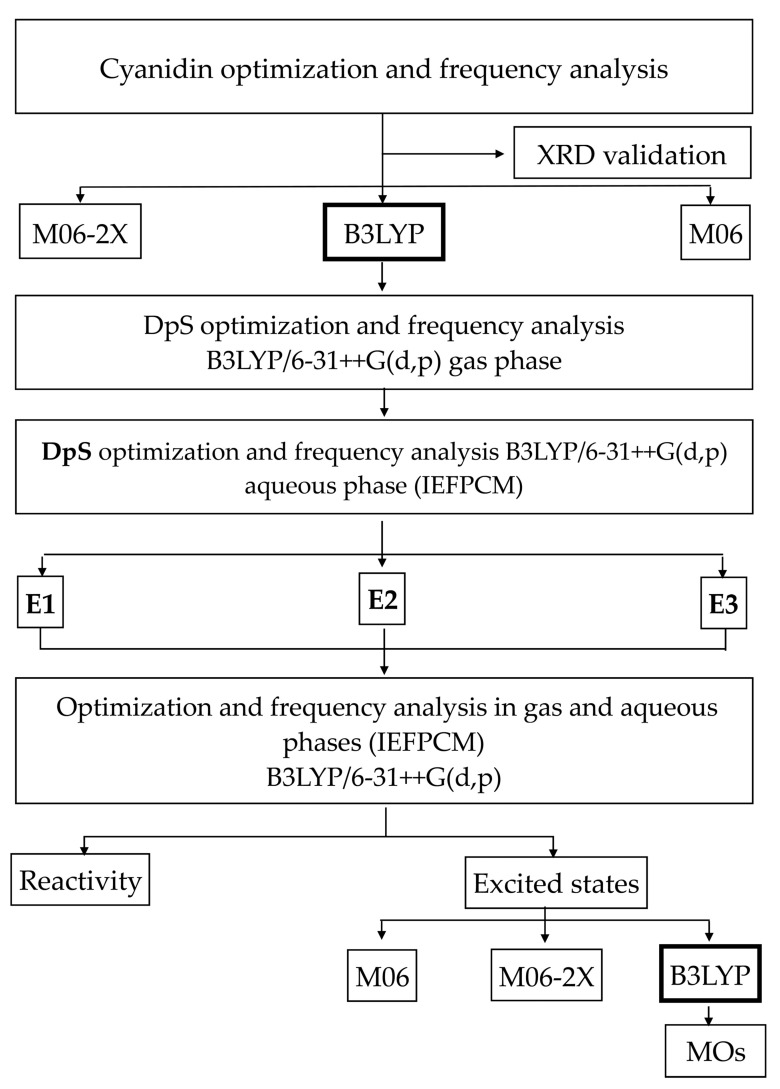
DTF and TD-DFT calculation scheme used to characterize DpS and its esters. Functional showing the best correlation is in a bold chart.

Gaussian16 program was employed to compute all the theoretical calculations [39]. The complete methodology is summarized in Figure 8.

## 4. Conclusions

Density Functional Theory gives a useful model when trying to obtain information about molecular behavior, which cannot be fully characterized by experimental procedures. Particularly in this work, DFT: B3LYP/6-31++G(d,p) presents the best correlation for the geometrical structure parameters, while the TD-DFT: B3LYP/6-31++G(d,p) methodology gives a view of the quantic landscape of the electronic behavior of lipophilized anthocyanins. 

B3LYP is a high correlation functional, which describes better the MOs involved in the UV-Vis spectra of the anthocyanin-esters due to the conjugated nature of the flavylium moiety. For instance, it shows that neither of the esters contribute to the HOMO and LUMO. Although, the esterification of the anthocyanins may slightly compromise the electron resonance along the flavylium core, changing the transition at the λ_calc_ and the number of transitions in the excited state with the strongest oscillator strength.

The reactivity in anthocyanin-esters could change from the anthocyanin precursor if the lipophilization occurs in the flavylium core. E3 maintains the same excited states and electronic transitions as the DpS. From all tested methodologies, theoretical results of the E3 at TD-DFT: B3LYP/6-31++G(d,p) demonstrated that the lipophilization in the sambubioside fragment prevents the loss of absorbance intensity in the anthocyanin-esters; since its excited states remained the same as its precursor DpS. For this reason, the lipophilization of anthocyanins in the sugar moiety could improve the solubility in lipidic systems without significantly diminishing their color.

Although DFT is a reliable method, the exploration of the potential energy hypersurface is a complex numerical problem. The challenge involved in the ground state calculation is further incremented when adding the complexity of the resonant structure of the flavylium cation. Also, TD-DFT is known to have a good response when the excited structure does not deviate far from the ground state structure, and the chosen functional is adequate for the system under study. These considerations contribute to understanding the result that B3LYP functional is the one that better reproduces the experimental maximum wavelength λ_exp_.

Another strength of the study, is the use of water as solvent in the calculations in order to mimic real experimental conditions. Lastly, one of the main contributions of this work is that experimental data was used to assist in the building of the molecular model, using DFT based quantum-chemical methods, meaning that mass spectrometry fragmentation data gave the information of the possible position of the esterification.

## Figures and Tables

**Table 1:** Bond lengths of cyanidin (gas phase) calculated with different methodologies and compared with experimental data reported by Ueno [28].

	Base 6-31++G(d,p)			
Distances * (Å)	Experimental	B3LYP	M06-2X	M06
C2-C1′	1.453	1.451	1.453	1.427
C4a-C5	1.432	1.432	1.429	1.411
C1′-C2′	1.409	1.417	1.411	1.413
C6-C7	1.413	1.418	1.417	1.401
C2-C3	1.396	1.408	1.402	1.410
C3′-C4′	1.400	1.415	1.411	1.394
C4-C4a	1.382	1.399	1.396	1.404
C6′-C1′	1.404	1.411	1.402	1.415
C4a-C8a	1.408	1.421	1.414	1.393
C7-C8	1.387	1.398	1.392	1.427
R^2^	-	0.9329	0.9185	0.9193

* Structure is shown in Figure 1.

**Table 2:** Vertical absorption wavelength (λ_calc_), oscillator strength (*f*) and main electronic transitions for DpS, E1, E2 and E3, calculated with the B3LYP/6-31++G(d,p) level of theory in aqueous phase, employing the IEFPCM model ^(a)^.

Molecule		λ_calc_ (nm)	Oscillator Strength (*f*)	Electronic Transitions		
**DpS**	1	487.7	0.5409	H-0→L+0 (+92%)	H-2→L+0 (+7%)	
	2	463.0	0.0273	H-1→L+0 (+98%)		
	3	422.0	0.1237	H-2→L+0 (+89%)	H-0→L+0 (+8%)	
**E1**	1	484.0	0.0417	H-1→L+0 (+85%)	H-0→L+0 (+14%)	
	2	478.1	0.3598	H-0→L+0 (+76%)	H-1→L+0 (+15%)	H-2→L+0 (+8%)
	3	410.7	0.2969	H-2→L+0 (+89%)	H-0→L+0 (+9%)	
**E2**	1	485.4	0.0265	H-1→L+0 (+90%)	H-0→L+0 (+9%)	
	2	477.6	0.3769	H-0→L+0 (+81%)	H-1→L+0 (+10%)	H-2→L+0 (+8%)
	3	411.2	0.2943	H-2→L+0 (+89%)	H-0→L+0 (+9%)	
**E3**	1	486.9	0.5391	H-0→L+0 (+91%)	H-2→L+0 (+7%)	
	2	463.5	0.0280	H-1→L+0 (+99%)		
	3	422.4	0.1212	H-2→L+0 (+89%)	H-0→L+0 (+8%)	

^(a)^ Delphinidin 3-*O*-sambubioside λ_exp_ at 526 nm.

**Table 3:** Vertical absorption wavelength (λ_calc_), oscillator strength (*f*) and main electronic transitions for DpS, E1, E2 and E3, calculated with the M06/6-31++G(d,p) level of theory in aqueous phase, employing the IEFPCM model ^(a)^.

Molecule		λ_calc_ (nm)	Oscillator Strength (*f*)	Electronic Transition	
**DpS**	1	479.9	0.5886	H-0→L+0 (+93%)	H-2→L+0 (+7%)
	2	442.9	0.0137	H-1→L+0 (+99%)	
	3	407.8	0.1136	H-2→L+0 (+89%)	H-0→L+0 (+7%)
**E1**	1	470.4	0.4380	H-0→L+0 (+92%)	H-2→L+0 (+7%)
	2	458.7	0.0151	H-1→L+0 (+99%)	
	3	398.1	0.2720	H-2→L+0 (+89%)	H-0→L+0 (+8%)
**E2**	1	469.9	0.4380	H-0→L+0 (+92%)	H-2→L+0 (+7%)
	2	460.1	0.0167	H-1→L+0 (+99%)	
	3	398.5	0.2719	H-2→L+0 (+89%)	H-0→L+0 (+8%)
**E3**	1	469.9	0.4750	H-0→L+0 (+91%)	H-2→L+0 (+8%)
	2	465.5	0.0094	H-1→L+0 (+99%)	
	3	402.7	0.1793	H-2→L+0 (+87%)	H-0→L+0 (+8%)

^(a)^ Delphinidin 3-*O*-sambubioside λ_exp_ at 526 nm.

**Table 4:** Vertical absorption wavelength (λ_calc_), oscillator strength (*f*) and main electronic transitions for DpS, E1, E2 and E3, calculated with the M06-2X/6-31++G(d,p) level of theory in aqueous phase, employing the IEFPCM model ^(a)^.

Molecule		λ_calc_ (nm)	Oscillator Strength (*f*)	Electronic Transition		
**DpS**	1	436.9	0.6474	H-0→L+0 (+92%)	H-2→L+0 (+6%)	
	2	357.2	0.1095	H-1→L+0 (+50%)	H-2→L+0 (+39%)	
	3	350.3	0.0532	H-2→L+0 (+49%)	H-1→L+0 (+46%)	
**E1**	1	429.5	0.5450	H-0→L+0 (+93%)	H-2→L+0 (+6%)	
	2	373.5	0.0188	H-1→L+0 (+97%)		
	3	349.7	0.2484	H-2→L+0 (+86%)	H-0→L+0 (+5%)	
**E2**	1	428.9	0.5452	H-0→L+0 (+93%)	H-2→L+0 (+6%)	
	2	374.0	0.0182	H-1→L+0 (+97%)		
	3	349.8	0.2515	H-2→L+0 (+85%)	H-0→L+0 (+5%)	
**E3**	1	429.8	0.5582	H-0→L+0 (+91%)	H-2→L+0 (+7%)	
	2	378.1	0.0161	H-1→L+0 (+97%)		
	3	352.5	0.1833	H-2→L+0 (+83%)	H-0→L+0 (+7%)	H-4→L+0 (+5%)

^(a)^ Delphinidin 3-*O*-sambubioside λ_exp_ at 526 nm.
